# Direct Anterior Versus Posterior Approach in Total Hip Arthroplasty: A Literature Review Comparing Outcomes, Functional Recovery, and Return to Sports

**DOI:** 10.7759/cureus.111519

**Published:** 2026-06-25

**Authors:** Bartosz M Krukowski, Maciej Slysz, Michal Majewski, Ada Swiatko, Magdalena Zabczynska, Mateusz Chmiela, Weronika Janik, Anna K Czesyk

**Affiliations:** 1 Department of Orthopedics, Trauma and Hand Surgery, University Clinical Hospital, Wroclaw Medical University, Wrocław, POL; 2 Department of Oncological Surgery, Lower Silesian Oncology, Pulmonology and Hematology Center, Wrocław, POL; 3 Department of Surgery, Health Care Center, Oława, POL; 4 Department of Orthopedics, Trauma and Musculoskeletal Oncology, Voivodeship Specialist Hospital, Wrocław, POL; 5 Faculty of Medicine, Wroclaw Medical University, Wrocław, POL

**Keywords:** direct anterior approach, endoprosthesis, functional recovery, posterior approach, total hip arthroplasty

## Abstract

Hip osteoarthritis is becoming an increasingly serious and common problem. Global trends indicate a steadily rising number of people affected by this condition. At the same time, there is a gradually increasing need for treatment of this condition among younger demographic groups. This leads to an increasing need for research on the outcomes of total hip arthroplasty. The aim of this literature review is to compare the outcomes of procedures performed using the direct anterior and direct posterior approaches, taking into account an analysis of patients’ ability to return to full function and intraoperative parameters. The study is based on the results of studies, including meta-analyses and reviews, identified and selected using the PubMed, Cochrane, Embase, and Google Scholar databases. To ensure the appropriate quality of the analyzed studies and to present the current state of medical knowledge in the review, it was decided to apply exclusion criteria considering the publication date, objective, and methodological quality of the studies. Evidence from the analyzed studies indicates significant differences in the profile of complications, intraoperative parameters, and muscle damage between direct anterior access and posterior access. The findings suggest that the choice of approach does not affect residents’ learning curve. The studies demonstrated that regardless of the chosen approach, patients undergoing total hip arthroplasty can return to physical activity, which is a particularly important factor for young patients undergoing arthroplasty. Although both approaches constitute a safe standard of care for hip osteoarthritis, the analysis highlighted the ongoing need to study the impact of surgical approach selection on patients’ return to sports activities and postoperative pain management protocols. The choice of surgical technique should be based not only on the patient’s anatomy but also on their activity level.

## Introduction and background

Total hip arthroplasty (THA) is considered the global standard of care for the treatment of end-stage osteoarthritis (OA) of the hip. Despite the popularity of this method, the procedure is regarded as a last resort due to its invasiveness [1]. Given the steady increase in global use of hip implants, which continues to grow at a rate of 1.2% annually in Organization for Economic Co-operation and Development (OECD) countries [2], this represents a growing burden on the healthcare system. This trend is confirmed by international analyses predicting a significant and sustained increase in the burden associated with primary total hip arthroplasty across various healthcare systems by 2050 [3]. In recent years, there has been an increasing emphasis on studies aimed at monitoring parameters such as postoperative pain management, the speed of recovery after surgery, and the reduction of intraoperative blood loss [4]. The need for this data is particularly critical due to shifting demographic trends -- population-based studies indicate a disproportionate increase in the demand for THA procedures among younger age groups [5-7]. These patients have higher expectations regarding the restoration of mobility after surgery, as well as high-intensity physical activity [6,8,9]. This is also confirmed by trends in medical recommendations, which are becoming less restrictive for patients after THA [10]. These changes underscore the clinical importance of performing surgical procedures that minimize the negative impact on factors such as returning to a high level of activity after surgery [9].

At the same time, discussions regarding the optimal surgical approach for THA procedures are still ongoing. Currently, the most common surgical approaches in THA are the posterior approach (PA), direct anterior approach (DAA), and direct lateral approach (DLA) [11,12].

The main objective of this study is to conduct a comprehensive review of the literature comparing the posterior approach (PA) and the direct anterior approach (DAA). We recognize the importance of other surgical approaches in hip arthroplasty; however, out of concern for potential methodological heterogeneity resulting from comparing multiple, differing surgical approaches simultaneously, we decided to compare these two methods to ensure the best possible comparability of results. Expanding the review to include other approaches would require a broader, less focused analysis, given that many studies available online focus exclusively on comparing the outcomes of just two of the many existing surgical approaches.

Need for a review of current methods

Given the steadily increasing number of total hip arthroplasty (THA) procedures, it is crucial to conduct ongoing research that aggregates data on the quality and outcomes of these surgeries. Reviewing the parameters used to classify the leading surgical techniques available for THA is important, as it allows for the continuous updating of knowledge among specialists performing these procedures, tracking global trends in orthopedic surgery, facilitates the selection of the optimal procedure to ensure the best possible treatment outcomes for patients, and raises awareness regarding potential complications and long-term surgical outcomes.

This review not only facilitates rational treatment planning but also supports physicians in making clinical decisions based on the evidence-based medicine (EBM) paradigm, allowing for better selection of the surgical approach to achieve maximum patient satisfaction with the procedure. Furthermore, the study’s findings may support the process of structuring treatment algorithms.

## Review

Materials and methods

This paper provides a systematic review of the literature and scientific articles focusing on a comparative analysis of the course and outcomes of the THA procedure. As part of this review, scientific materials available online as well as in print were analyzed. To ensure an appropriate selection of studies, a set of exclusion criteria was established.

Data Sources

To ensure the appropriate selection of scientific studies for analysis, a review was conducted of materials available in academic databases, such as PubMed, Cochrane, Embase, and Google Scholar. The process of selecting relevant articles, which were then analyzed, was based on a review of titles and abstracts to conduct a preliminary assessment of the article's topic.

Search of the Literature

Our research began on April 3, 2026. The process of searching for works to include in this article, along with the simultaneous preparation of the publication, continued until May 1, 2026. We analyzed a total of 934 publications across four available content aggregators. Due to searching across multiple aggregators and employing a search strategy based on using general keywords searched separately, 356 duplicate works were eliminated during the initial screening phase. To ensure clarity of our research, we decided to present all screened results with the exact search bar phrase and the number of publications in Table 1. Search queries were tailored to each database's interface, which accounts for observed variations in retrieval numbers across platforms.

**Table 1:** Search phrases applied to electronic databases. DAA: direct anterior approach; PA: posterior approach.

Screened study phrase in search bar	PubMed	Google Scholar	Cochrane	Embase	All aggregators sum
Total hip arthroplasty approach	141	29	28	10	208
Direct anterior approach posterior approach	245	10	10	0	265
Total hip arthroplasty surgical approach	112	0	0	0	112
DAA PA	107	0	0	0	107
Total hip arthroplasty approach outcomes	77	0	0	0	77
Direct anterior approach posterior approach meta analysis	50	5	0	0	55
Total hip arthroplasty opioid approach	30	10	10	0	50
Total hip arthroplasty pain approach	20	10	0	0	30
Return to sports total hip arthroplasty	30	0	0	0	30

For the purpose of data extraction, a custom template was developed to ensure consistency in the analysis of all included studies. The following information was systematically collected: study characteristics (authors, year, project, sample size); intervention details (DAA vs. PA); surgical approaches analyzed in the reviewed study; results analyzed in the study, such as operative time, blood loss, complication rates, revision rates, and other parameters considered in this literature review. For studies with incomplete data, the analysis relied on available abstracts or the final results section. Any discrepancies regarding the interpretation of data and the quality of the selected studies were discussed with the co-authors to ensure the desired quality of the review.

We then proceeded to the process of excluding works. The study selection process was conducted in accordance with the Preferred Reporting Items for Systematic Reviews and Meta-Analyses (PRISMA) guidelines and comprised a structured, two-stage screening protocol. To optimize the process, the first stage involved excluding studies based on an analysis of their titles. During this process, we focused on selecting publications that directly addressed DAA and PA. In this stage, 477 studies were eliminated. 

After eliminating studies based on title analysis, we analyzed the abstracts of the remaining studies in the second stage, focusing on the reporting of relevant clinical and functional parameters. At this stage, we excluded 36 studies.

The 65 studies we selected after analyzing the titles and abstracts were then evaluated for eligibility. It was crucial for us to screen the publications based on their inclusion in meta-analyses that had also been qualified in the previous stage. We then focused on selecting the appropriate materials using the exclusion criteria listed in the next subsection.

Exclusion Criteria

To ensure the selection of publications that most closely correspond to current medical knowledge, it was decided to exclude from the selection process studies published before 2010. Studies that did not focus on comparing outcomes of procedures using DAA and PA approaches were excluded. If a study contained data on surgical outcomes for only one of these approaches, it was excluded, as well as studies of low methodological quality. The quality of publications was evaluated using the Newcastle-Ottawa Scale (NOS) [13]. Review articles and meta-analyses were excluded from the formal risk-of-bias assessment as NOS is not applicable to these publication types. Studies published in languages other than English and Polish were also excluded. Ultimately, we also decided not to include the results of studies that were included in comparative meta-analyses. Instead, we chose to refer to the entire analysis, which made it easier to synthesize the results from multiple studies simultaneously.

After reviewing the available studies and excluding those meeting the above criteria, the studies were categorized based on their research outcomes and the parameters being compared to systematize the structure of this publication.

**Figure 1:**
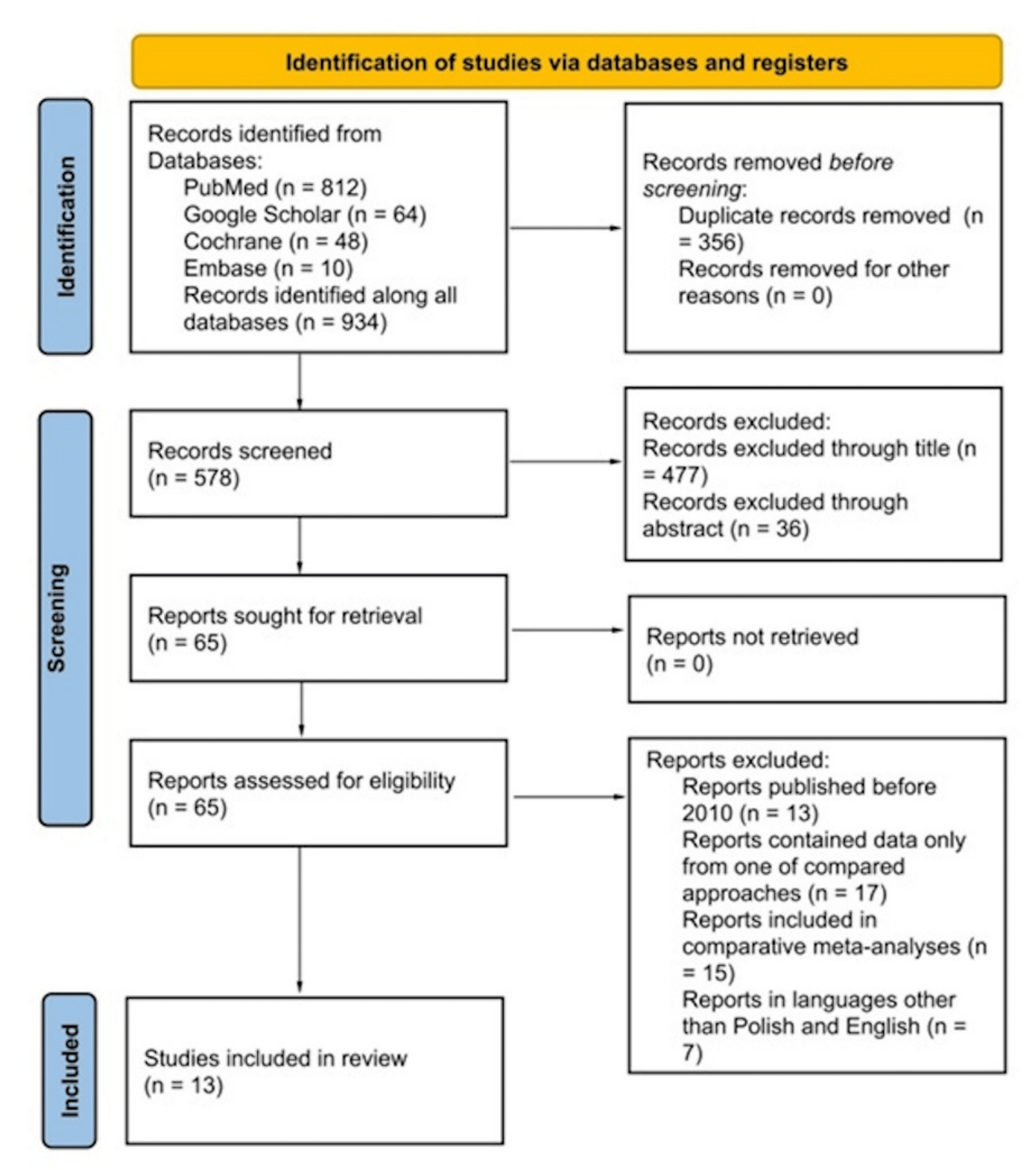
PRISMA diagram. PRISMA: Preferred Reporting Items for Systematic Reviews and Meta-Analyses.

As a result of the search for the analyzed studies and the selection process, we identified 13 studies on which we based our review. We have presented the process and details of the publication selection in Figure 1, which shows our PRISMA diagram [14]. Table 2 includes details of each publication included in our review.

**Table 2:** Final publications included in review based on PRISMA diagram. *Data included in meta-analysis. N/A: not applicable: The numerical value has been omitted for meta-analyses because these studies include aggregated data from multiple publications, and the sample size varies depending on the parameter being analyzed. N/A in NOS score column: not applicable for this study design. DAA: direct anterior approach; PA: posterior approach; LA: lateral approach; AA: anterior approach; PRISMA: Preferred Reporting Items for Systematic Reviews and Meta-Analyses.

Author and publication year	Article title	PMID	Study design	Total sample size	Sample size (DAA/AA)	Sample size (PA)	Study limitations	Main outcomes	Newcastle-Ottawa Scale (NOS) Score
Gulbrandsen et al. 2022	Total hip arthroplasty: direct anterior approach versus posterior approach in the first year of practice	35821938	Retrospective cohort study	181	91	90	Retrospective study design; Cases were reviewed from a single academic center, which limits the generalizability of the findings; Additionally, the specific approaches were performed by separate surgeons. Small sample size; Difference between mean BMI between cohorts.	Similar complication rates. PA faster (99 vs. 111 min). DAA showed significantly lower revision rate. No significant difference in learning curve progression between approaches.	8/9
Soza et al. 2021	A comparative of a single novice surgeon’s direct anterior approach and posterior approach learning curves in total hip arthroplasty: a retrospective cohort study	34129119	Retrospective cohort study	376	187	184	Retrospective study design; Demographic differences between the two groups compared	Comparable safety and outcomes. DAA shows benefits in length of stay and dislocation rates, with similar complication and surgical time profiles.	8/9
Mead and Bugbee 2022	Direct anterior approach to total hip arthroplasty improves the likelihood of return to previous recreational activities compared with posterior approach	34982059	Retrospective cohort study	200 (130 included)	100 (66 included)	100 (64 included)	Retrospective study design; Lack of data on preoperative recreational activities; Consequently, the authors could not determine whether the difference in recreational profile was related to the surgical approach or the patients' preoperative inclinations	DAA shows advantage in return-to-sport metrics (pre-operative activities and any post-THA sports) compared to PA. Walking remains the most popular post-operative activity.	7/9
Lin et al. 2025	Return to sports after total hip arthroplasty: patterns of participation and sport-specific outcomes	41248747	Retrospective cohort study	1115	556	519	Retrospective study design; Short follow-up; Heterogeneity of procedures; Potential confounding factors-patient-reported outcomes.	No statistically significant difference in return-to-sport rates between DAA and PA (68.1% vs 77.0%). Overall high rate of return to sports post-THA observed.	8/9
Svarnas et al. 2025	Postoperative muscle atrophy and fatty degeneration with respect to surgical approaches in total hip arthroplasty	40056179	Retrospective cohort study	493	145	107	Retrospective study design; Limitations related to imaging; Demographic and methodological limitations; Multiple included surgeons.	Muscle changes seen in both approaches (gluteus minimus, iliopsoas damaged). DAA advantage: no gluteus medius fatty degeneration. PA disadvantage: higher external rotator damage.	8/9
Liu et al. 2025	Comparative efficacy of direct anterior approach versus conventional surgical approaches in total hip arthroplasty: a systematic review and meta-analysis of randomized clinical trials	41013681	Systematic review and meta-analysis	1575*	N/A	N/A	Heterogeneity of procedures; Potential selection bias; Variations in patient demographics, surgeon expertise, and operative techniques across included studies; Incomplete complication coverage	DAA is associated with significantly longer mean procedure duration compared to conventional approaches (including PA). No statistically significant difference in intraoperative blood loss.	N/A
Ang et al. 2023	Comparing direct anterior approach versus posterior approach or lateral approach in total hip arthroplasty: a systematic review and meta-analysis	37010580	Systematic review and meta-analysis	2010 *(792 DAA vs PA)*	N/A	N/A	Heterogeneity of procedures; Potential selection bias; Potential confounding effect of surgeon experience; Patient heterogeneity	Meta-analysis confirms DAA requires significantly longer operative times compared to PA, allowing for a direct and accurate comparison between these two approaches. Shorter hospital stay in the DAA group. No statistically significant differences in complication rates between DAA and PA.	N/A
Singh et al. 2022	Similar outcomes achieved between anterior and posterior approach total hip arthroplasty using dual mobility implants	35821937	Retrospective cohort study	495	55 (AA)	440	Retrospective study design; Patient heterogeneity; Sampling imbalance; Implant heterogeneity; Protocol variability - institutional protocol changes between 2011 and 2021 may have influenced outcomes over the study period; As the study was conducted at an orthopedic specialty hospital, findings may not apply to low-volume surgeons or those less experienced with the chosen surgical approaches.	No statistically significant difference in procedure duration.	8/9
Shen et al. 2023	Direct anterior approach provides superior prosthesis adaptability in the early postoperative period of total hip arthroplasty	36513388	Retrospective cohort study	376	41	335	Sampling imbalance; Retrospective study design; Significant heterogeneity surrounded the rehabilitation programs after surgery; Gait abnormalities and lifestyle factors not controlled.	Comparable operative time and intraoperative blood loss between DAA and PA.	7/9
Ghandour et al. 2025	Direct anterior approach versus posterior approach in total hip arthroplasty: a systematic review and meta-analysis	40607318	Systematic review and meta-analysis	44477*	N/A	N/A	Potential selection bias; High heterogeneity; variation in follow-up intervals;	Results regarding incision length in favour of DAA.	N/A
Seah et al. 2019	Postoperative opioid consumption after total hip arthroplasty: a comparison of three surgical approaches	31311666	Retrospective cohort study	560	179	381 (LA and PA)/203 (PA)	Retrospective study design; Influence of individual pain tolerance;	DAA is associated with lower postoperative opioid consumption compared to PA.	7/9
Streck et al. 2025	Patient-reported outcomes during the first month following anterior and posterior total hip arthroplasty and hip resurfacing	41387849	Retrospective cohort study	207	126	59 PA + 21 Hip Resurfacing	Retrospective design; Unequal group sizes; Inherent demographic differences between groups; Lack of matching; reliance on patient-reported data; Variability in implants used.	No statistically significant difference in postoperative opioid consumption between DAA and PA.	7/9
Hoskins et al. 2024	A comparison of cemented femoral fixation via anterior versus posterior approach total hip arthroplasty: an analysis of 60,739 total hip arthroplasties	38529902	Retrospective cohort study	60739	10742	49997	Retrospective study design; Lack of radiological data; Surgeon training/experience/volume not recorded; Learning curve effect unknown; potential confounding by spinal pathology/previous surgery; Registry only records revisions (non-revision complications missed); Lack of periprosthetic fracture classification; Short follow-up;	Comparable overall revision rates between DAA and PA. DAA associated with higher revision rates for femoral component loosening, but lower revision rates for infection and dislocation. No significant difference in revision rates due to periprosthetic fractures.	7/9

Results

Learning Curve and Outcomes of Orthopedic Residents

From the perspective of large clinical centers that train resident surgeons, initial outcomes and the learning curve for a given medical procedure may be important factors influencing treatment outcomes. The publication focusing on results of outcomes for such approaches within the first year of practice after fellowship [15] revealed several important findings regarding the performance of surgeons early in their independent practice. We have included some of the parameters we considered particularly significant in Table 3.

**Table 3:** Surgical outcomes and complication rates of resident-performed total hip arthroplasty: direct anterior vs. posterior approach. *p-value is considered significant (p<0.05). Continuous data were analyzed using an unpaired, two-tailed t-test. Categorical variables, including complication rates, were compared between groups using the Chi-square test or Fisher’s exact test, as appropriate [15]. DAA: direct anterior approach; PA: posterior approach.

Parameter	DAA	PA	p-value
Cases	91	90	
Computed total blood loss (mL)	1109	1260	0.06
Infections	3	3	0.64
Nerve-related complications	3	0	0.4
Procedure duration (min)	111	99	0.001*
Dislocations	1	4	0.17
Reoperations	1	3	0.31
Revisions	0	5	0.04
Intraoperative femoral fractures	2	1	0.48
Postoperative periprosthetic fractures	2	3	0.64

An analysis of surgical complication rates showed similar percentages in both groups due to the absence of statistically significant differences, including the rates of infections, dislocations, and periprosthetic fractures. However, a significant difference was found in the duration of the operation in favor of the posterior approach, which was significantly faster to perform, with a mean duration of 99 minutes compared to 111 minutes for the direct anterior approach (p = 0.001). The study also noted a trend toward shorter procedure times in both compared surgical techniques; however, no statistically significant differences in the progression curve were demonstrated between direct anterior and posterior approaches. This difference was observed only when comparing the first 50 procedures performed using each approach. The final statistically significant difference that caught the researchers’ attention was the number of revisions, which was statistically significantly lower for the DAA approach. Ultimately, the study suggests that the learning curve is not unique to any single specific approach.

These findings were confirmed in another study [16], whose authors noted that even at an early stage in the training of orthopedic surgeons, both approaches are safe and their outcomes are acceptable to patients. It was noted that the DAA does not present a higher complication rate with regard to periprosthetic fractures or the incidence of infection, while ensuring a shorter hospital stay and a lower rate of dislocations. The duration of the procedure after a certain number of surgeries was comparable for both approaches.

Return to Sport Activities

Given the evolving demographic trends among patients undergoing total hip arthroplasty (THA) [5-7], a return to sports activities may be a very important factor that contributes positively to mental health outcomes and subjective life satisfaction [17]. Patients with hip osteoarthritis are often limited in their ability to participate in sports prior to hip replacement surgery. Studies have shown that physical activity levels increase after THA compared with pre-surgery levels [18]. Patient satisfaction may be influenced not only by a return to the sports they practiced before hip replacement surgery, but also by any opportunity to engage in physical activity. Statistics indicate that walking is the most common form of recreation [19].

In terms of returning to sports activities after THA, the DAA has been shown to have an advantage [20]. In the group of patients operated on via the anterior approach, the statistics regarding a return to the sports activities practiced before surgery, as well as those indicating the practice of any sport after THA, were more favorable than in the group of patients operated on via the posterior approach. The study showed that among patients who underwent THA, walking was also the most popular form of physical activity practiced after surgery.

Contrary to these findings, a study based on a retrospective analysis of patients who underwent THA assessed their return to sports activities one year after surgery [21]. It did not reveal any statistically significant differences in the return to sports activity between the two groups. The rates of return to sports in the study were 68.1% and 77.0%, respectively. However, the researchers highlighted a significant finding consistent with the results of other studies--the rate of return to sports after THA is high, and most patients continue their sports activities after surgery.

When analyzing the results of the above studies, it is not possible to unequivocally conclude that one of the approaches analyzed is superior. Although the study conducted by Mead and Bugbee [20] suggests an advantage for the DAA, the study by Lin et al. [21] indicates no statistically significant difference in return to sports among patients who underwent THA via the direct anterior and posterior approaches. This discrepancy may indicate that other factors, such as rehabilitation protocols or patient motivation, may play an equally important role in the process of returning to sports activities.

Muscle Atrophy and Fatty Degeneration

Soft tissue damage is a significant factor affecting a patient’s return to function after THA. Both muscle damage and soft tissue degeneration can affect limb function after surgery [22]. It has been demonstrated that both approaches compared in this publication cause changes in the muscles surrounding the hip joint. The study did not identify a technique with unequivocally superior outcomes for the patient; however, a significant finding is that the DAA was the only one characterized by the absence of fatty degeneration of the gluteus medius muscle.

The study showed that the posterior approach has a more negative impact on the external rotator group. At the same time, it was demonstrated that damage to this muscle group also occurred with the direct anterior approach. Damage to the gluteus minimus and iliopsoas muscles was noted with both approaches. 

Knowledge of the varied effects of different approaches on the hip musculature provides important information that facilitates the decision-making process regarding the selection of the appropriate approach during THA surgery, which may lead to better postoperative outcomes and the adaptation of the technique to the patient’s needs.

Intraoperative Parameters and Length of Hospitalization

A meta-analysis of data comparing selected measurable parameters revealed a statistically significant difference to the disadvantage of the DAA approach over conventional approaches [23]--a group that also included the posterior approach--in terms of mean procedure duration, indicating a shorter procedure duration (mean difference (MD) = 14.5, 95% CI: 9.14-19.86, p < 0.01). At the same time, no statistically significant difference was found in the amount of intraoperative blood loss. It should also be emphasized that PA was not the only access method in the group compared to DAA. The results of this study also correlate with another meta-analysis [24], which confirmed that procedures performed via an anterior approach were characterized by longer operative times (MD = 17.38 min, 95% CI: 12.28-22.47, P < 0.001)--in this case, DAA results were directly compared with PA, allowing for a more accurate comparison of these two approaches.

Data from other available studies analyzed for this work also provide information regarding these parameters. No statistically significant difference in procedure duration was found for surgeries using dual-mobility implants [25]. Furthermore, a study focusing on prosthesis adaptability--which was not included in the analysis cited at the beginning of this subsection--did not show statistically significant differences in operative time or intraoperative blood loss between DAA and PA [26].

It is also worth noting the results regarding incision length. The authors of the meta-analysis that considered this parameter [27] noted that it favors DAA (MD = −33.75 mm, 95% CI -42.97 to −24.54, p = 0.001), along with a shorter hospital stay (MD = −0.31 days, 95% CI -0.55 to −0.07, p = 0.01) [24]. In a study conducted by Gulbrandsen et al., no statistically significant differences in length of stay were observed among residents in training [15]. However, a study by Soza et al. [16] indicates that patients who underwent the procedure via direct anterior access had a shorter average hospital stay. Length of hospital stay was 4.54 days for the DAA group and 5.53 days for the PA group (p-value < 0.001).

Pain and the Postoperative Use of Opioids

One of the factors influencing patient satisfaction with the procedure is not only postoperative hip function itself, but also the pain associated with the procedure and its aftermath, as well as the need for analgesics. Orthopedic patients are a group at risk of severe pain during the postoperative period [28]. Good outcomes of analgesic treatment in the postoperative period correlate with better functional outcomes during rehabilitation [29].

It has been demonstrated that the anterior approach is associated with lower opioid consumption in the postoperative period compared to PA [30]. It has also been shown that pain decreased proportionally for both approaches. The factors with the greatest influence on patients’ need for opioids were age and opioid naivety. It is also worth considering the findings of a meta-analysis comparing the outcomes of DAA procedures with those performed using other approaches, including PA [23]. The authors note a reduction in pain intensity in the first few days after surgery in favor of DAA compared with other approaches. Meta-analysis comparing the direct anterior approach with the posterior and lateral approaches [24] suggests that the lower incidence of pain associated with the anterior approach may be due to minimal soft-tissue trauma.

However, the literature does not reach a full consensus regarding the conclusions drawn from studies on opioid consumption. Contrary to these findings are the results of a study conducted by Streck et al. [31]. No statistically significant differences in opioid consumption were found between the DAA and PA groups. This discrepancy in results suggests that the choice of approach during THA may have less impact on a patient’s analgesic consumption profile than appropriately selected perioperative care protocols, such as the Enhanced Recovery After Surgery (ERAS) protocol [32].

Complication Rates and Revisions

In terms of medical complications, a meta-analysis of data from available studies did not reveal any statistically significant differences between DAA and PA [24]. Both methods present a comparable risk of periprosthetic fractures, dislocations, and venous thromboembolic complications. 

When comparing the outcomes of DAA and PA in cemented femoral fixation [33], the revision rate for DAA was not significantly higher than that for PA. However, it was noted that the profile of revisions due to various complications differed depending on the access method used. The percentage of surgeries due to periprosthetic fractures did not differ in a statistically significant manner between DAA and PA. DAA, however, was significantly higher than PA in terms of the revision rate due to femoral component loosening and significantly lower than PA in the case of revisions due to infection and dislocation.

The results for residents in training in the Gulbrandsen study (2022) showed no statistically significant differences in the number of dislocations, periprosthetic fractures, infections, complications, reoperations, or complications related to nerve damage [15]. However, a statistically significantly lower revision rate was observed in the DAA group. A study by Soza et al. [16] also indicated that DAA is not associated with a higher complication rate than PA, neither in terms of infection rates nor in the case of periprosthetic fractures. 

Discussion

Current scientific reports indicate some significant differences between DAA and PA. Residents’ training in the procedure appears to yield similar results in terms of improving intraoperative parameters as the number of procedures performed increases [15]. At the same time, both techniques exhibit comparable complication rates, although a statistically significantly lower number of revisions was reported for DAA. Although the duration of the procedure, despite the heterogeneity of the results of the analyzed studies [23,25], may suggest an advantage for PA, no statistically significant differences were observed in blood loss and incision length [27], and the length of hospitalization indicates an advantage for DAA [24].

In the context of patients leading an active lifestyle, it is worth noting that DAA is characterized by the absence of fatty degeneration and a different profile of muscle damage [22]. This may constitute an important argument in the decision-making process regarding the performance of THA in younger patients, who may constitute a larger age group in the future [5-7]. DAA may allow them to return more efficiently to the sports activities they engaged in prior to THA [20], which has a positive impact on mental well-being and life satisfaction [17]. However, positive outcomes regarding the return to sports activities can ultimately be achieved in any group of patients who have undergone THA, regardless of surgical access [21]. Based on the findings of the two publications cited in this paper, there is no clear scientific consensus confirming the superiority of DAA over PA in terms of patients’ return to sports activities [20,21].

Although the analyzed studies do not allow for a consensus regarding which approach results in lower opioid use in the postoperative period, some studies lean toward conclusions suggesting greater pain and, consequently, a higher need for opioids in the PA group [23,24,30,31]. It is important to continue research on the proper implementation of postoperative care methods in patients undergoing THA, such as the ERAS protocol [33].

Studies indicate that both PA and DAA are characterized by a comparable incidence of complications [24]; however, the complication profile itself may vary depending on the access method used, as demonstrated by studies on cemented femoral fixation [32].

Ultimately, although both methods constitute a safe standard of care for hip osteoarthritis, awareness of the differences in their complication profiles, along with appropriate pain management and an understanding of the patient’s needs and lifestyle prior to deciding which approach to choose, may lead to better treatment outcomes. To objectify the decision-making process regarding the personalized selection of surgical approach for the purpose of maximizing the benefits of the procedure, further prospective studies are necessary. Further research on the return to fitness enabling participation in sports activities may prove particularly valuable in the context of changes in patient demographics [5-7].

Limitations

Given the limited number of scientific studies directly comparing the outcomes of surgery using direct anterior access versus posterior access, this scoping review is based on a limited number of publications. The greatest difficulty in gathering information for this study was finding publications that directly compared DAA with PA. Due to the wide variety of approaches in hip arthroplasty, there are many publications comparing the outcomes of surgeries using different approaches; however, we considered it inappropriate to include publications that do not directly compare the outcomes of the two approaches being compared.

## Conclusions

The choice of the appropriate surgical approach should be based on the individual needs of patients. The complication profile may differ between DAA and PA, although their incidence is similar for both groups. Muscle damage and fatty degeneration differ between the two approaches, which may be particularly important for patients leading an active lifestyle. Both approaches are characterized by a similar learning curve for residents. Based on studies analyzing pain and opioid use, there are no unanimous conclusions; however, some studies indicate better outcomes with the DAA. There is a need for further research focusing on the appropriate selection of surgical approach based on lifestyle and expected THA outcomes in specific patient groups, particularly given changing demographic trends. Some studies have shown that DAA may result in a higher rate of return to sports, although a high rate of return to sports was observed in every group of patients who underwent THA.
